# Supplementary figures and images for: Complex Contagion of Campaign Donations
Source: PLoS One. 2016 Apr 14;11(4):e0153539. doi: 10.1371/journal.pone.0153539 (PMC4831683; doi:10.1371/journal.pone.0153539)

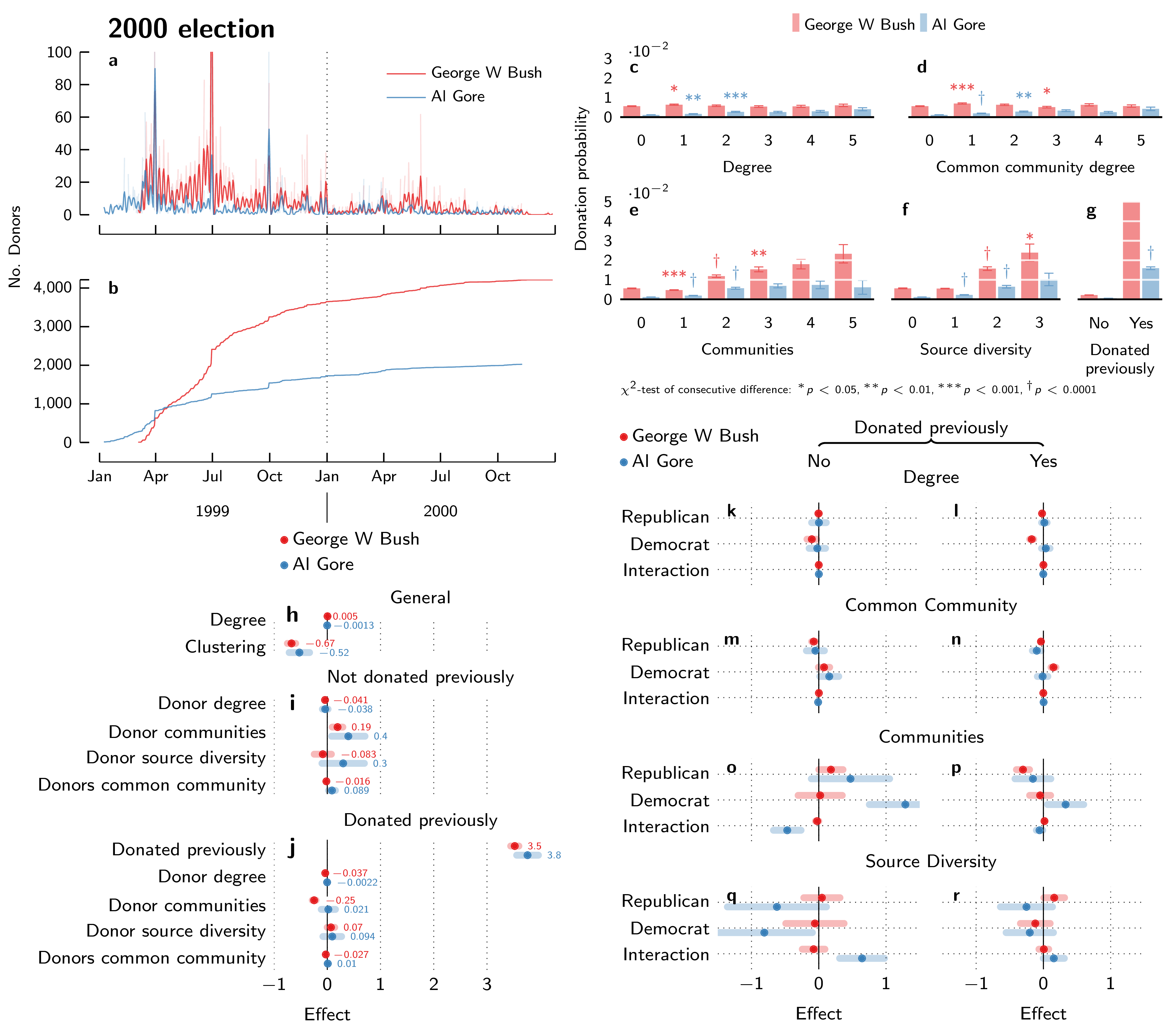

Supplement: S1 Fig — Daily donors (a)—raw data is transparent, smoothed data is solid—and cumulative donors (b), probability effect of (c) donor degree, (d) common community donor degree, (e) donor communities, (f) source diversity and (g) previous donation. Logistic regression results (h) general effects, (i) network effects for new donors, and (j) network effects for old donors. Logistic regression result for cross-cutting effects, distinguished by old/new donors for donor degree (k)–(l), common community donor degree (m)–(n), donor communities (o)–(p), and source diversity (q)–(r). Error bars show 95% confidence intervals for the coefficients. (TIFF) [file pone.0153539.s001.tiff]

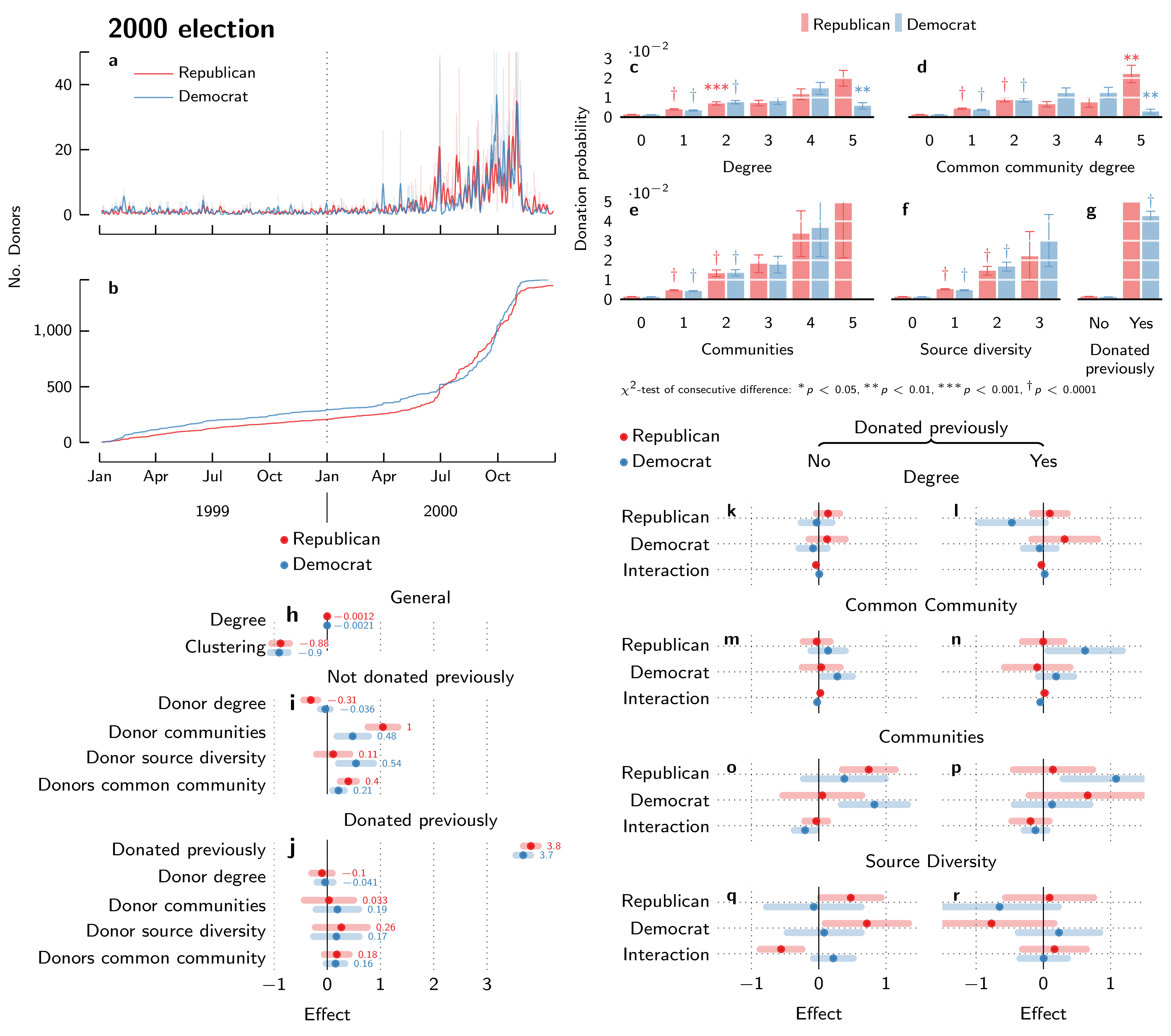

Supplement: S2 Fig — Daily donors (a)—raw data is transparent, smoothed data is solid— and cumulative donors (b), probability effect of (c) donor degree, (d) common community donor degree, (e) donor communities, (f) source diversity and (g) previous donation. Logistic regression results (h) general effects, (i) network effects for new donors, and (j) network effects for old donors. Logistic regression result for cross-cutting effects, distinguished by old/new donors for donor degree (k)–(l), common community donor degree (m)–(n), donor communities (o)–(p), and source diversity (q)–(r). Error bars show 95% confidence intervals for the coefficients. (TIFF) [file pone.0153539.s002.tiff]

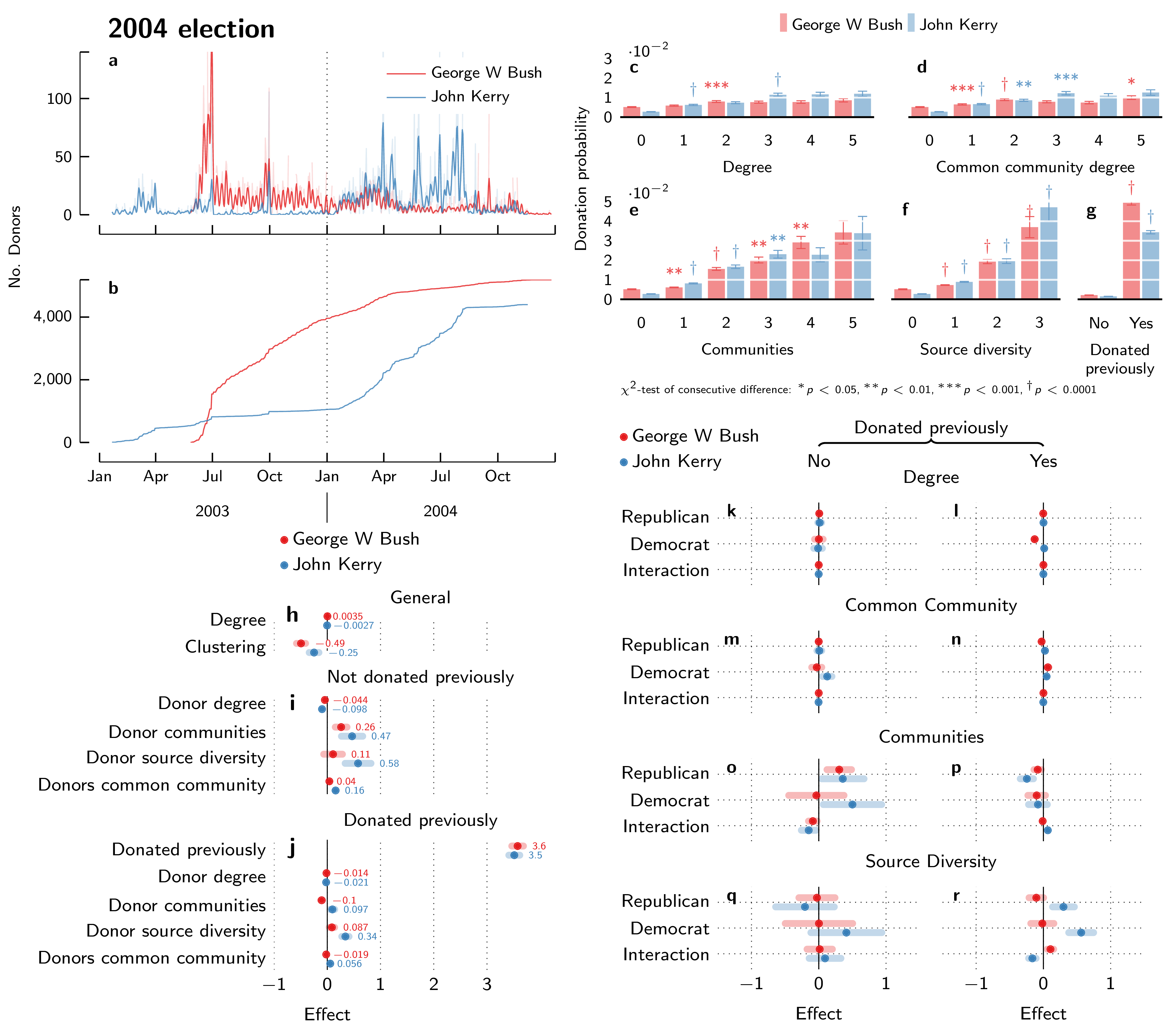

Supplement: S3 Fig — Daily donors (a)—raw data is transparent, smoothed data is solid— and cumulative donors (b), probability effect of (c) donor degree, (d) common community donor degree, (e) donor communities, (f) source diversity and (g) previous donation. Logistic regression results (h) general effects, (i) network effects for new donors, and (j) network effects for old donors. Logistic regression result for cross-cutting effects, distinguished by old/new donors for donor degree (k)–(l), common community donor degree (m)–(n), donor communities (o)–(p), and source diversity (q)–(r). Error bars show 95% confidence intervals for the coefficients. (TIFF) [file pone.0153539.s003.tiff]

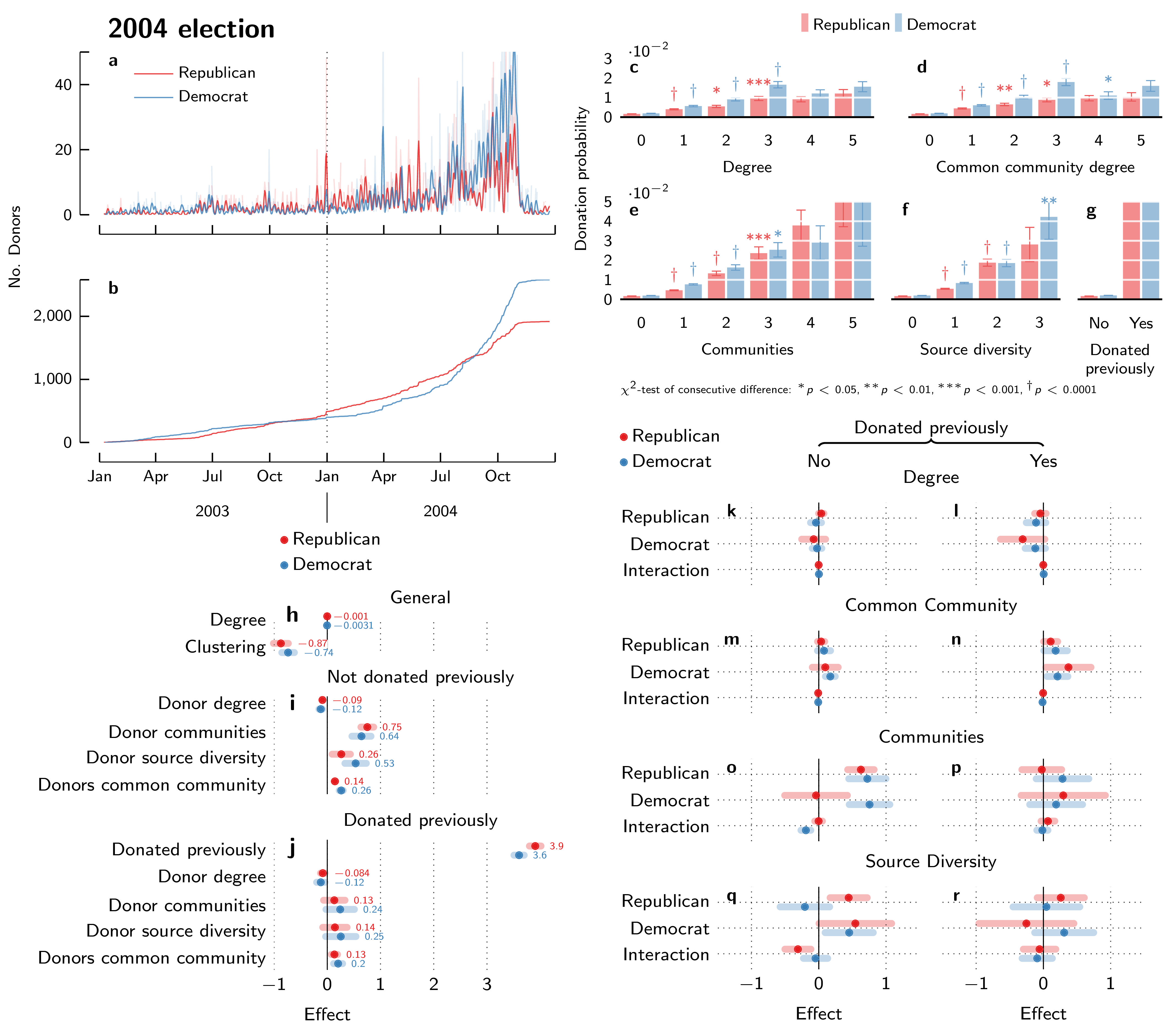

Supplement: S4 Fig — Daily donors (a)—raw data is transparent, smoothed data is solid— and cumulative donors (b), probability effect of (c) donor degree, (d) common community donor degree, (e) donor communities, (f) source diversity and (g) previous donation. Logistic regression results (h) general effects, (i) network effects for new donors, and (j) network effects for old donors. Logistic regression result for cross-cutting effects, distinguished by old/new donors for donor degree (k)–(l), common community donor degree (m)–(n), donor communities (o)–(p), and source diversity (q)–(r). Error bars show 95% confidence intervals for the coefficients. (TIFF) [file pone.0153539.s004.tiff]

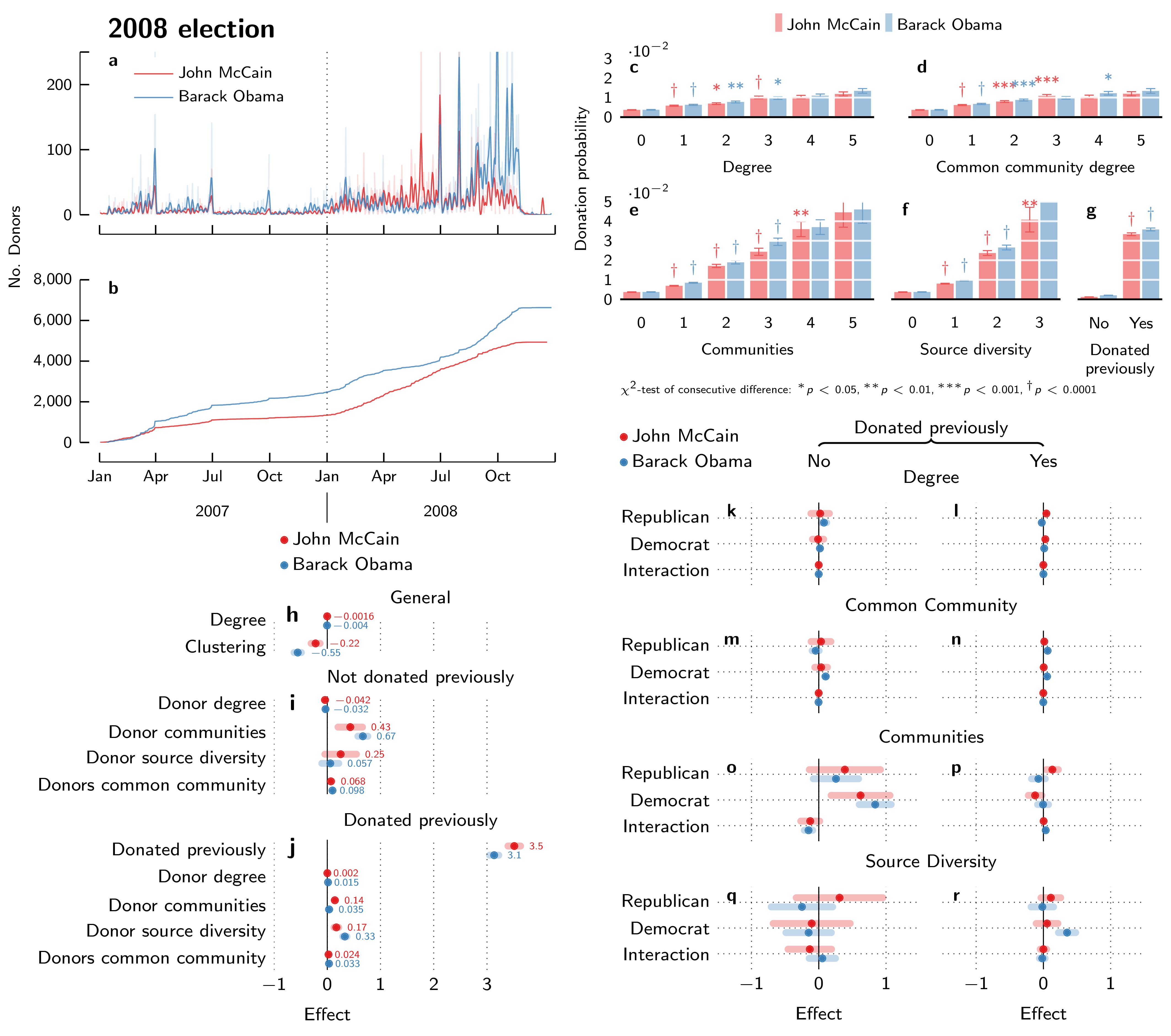

Supplement: S5 Fig — Daily donors (a)—raw data is transparent, smoothed data is solid— and cumulative donors (b), probability effect of (c) donor degree, (d) common community donor degree, (e) donor communities, (f) source diversity and (g) previous donation. Logistic regression results (h) general effects, (i) network effects for new donors, and (j) network effects for old donors. Logistic regression result for cross-cutting effects, distinguished by old/new donors for donor degree (k)–(l), common community donor degree (m)–(n), donor communities (o)–(p), and source diversity (q)–(r). Error bars show 95% confidence intervals for the coefficients. (TIFF) [file pone.0153539.s005.tiff]

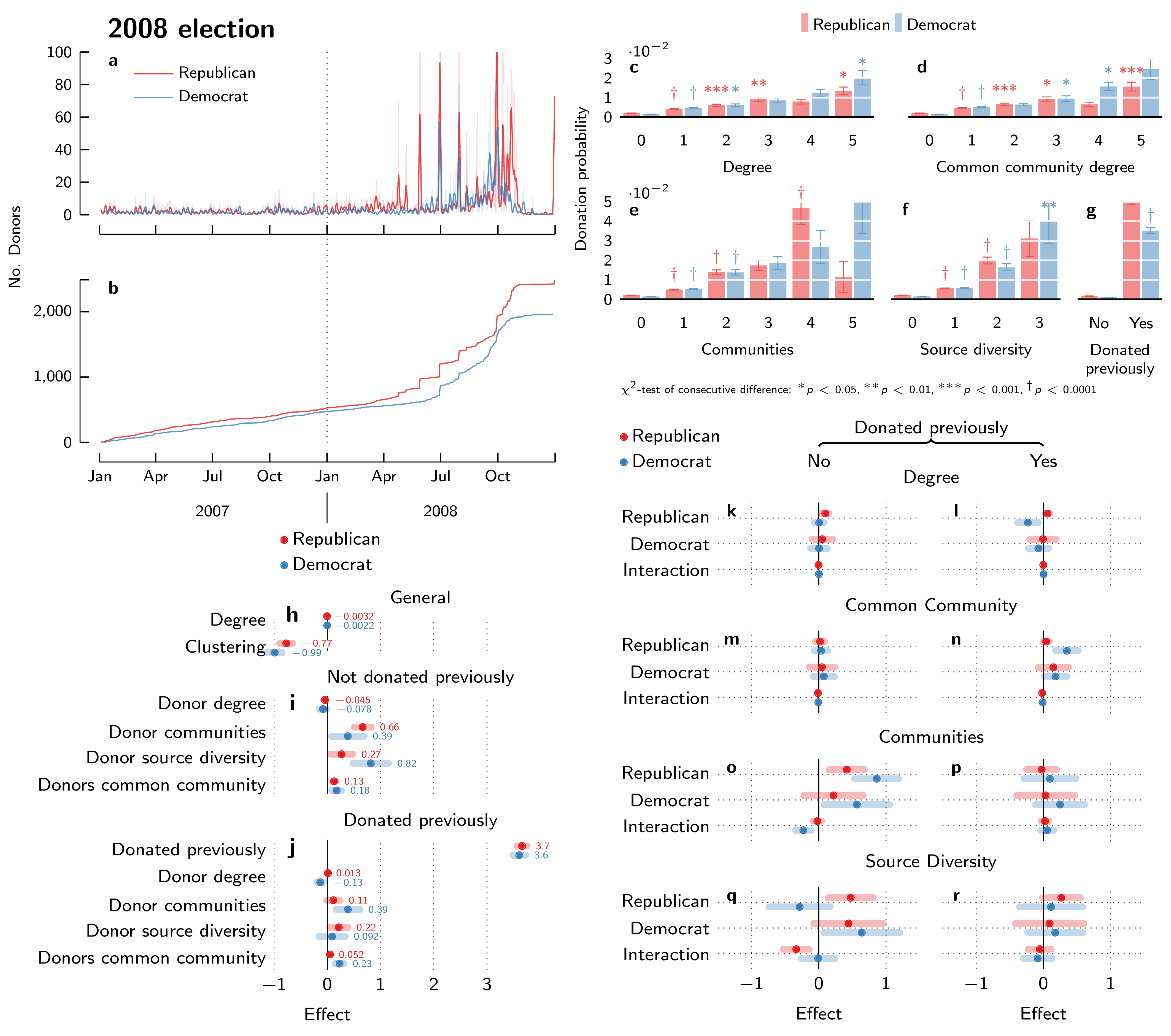

Supplement: S6 Fig — Daily donors (a)—raw data is transparent, smoothed data is solid— and cumulative donors (b), probability effect of (c) donor degree, (d) common community donor degree, (e) donor communities, (f) source diversity and (g) previous donation. Logistic regression results (h) general effects, (i) network effects for new donors, and (j) network effects for old donors. Logistic regression result for cross-cutting effects, distinguished by old/new donors for donor degree (k)–(l), common community donor degree (m)–(n), donor communities (o)–(p), and source diversity (q)–(r). Error bars show 95% confidence intervals for the coefficients. (TIFF) [file pone.0153539.s006.tiff]

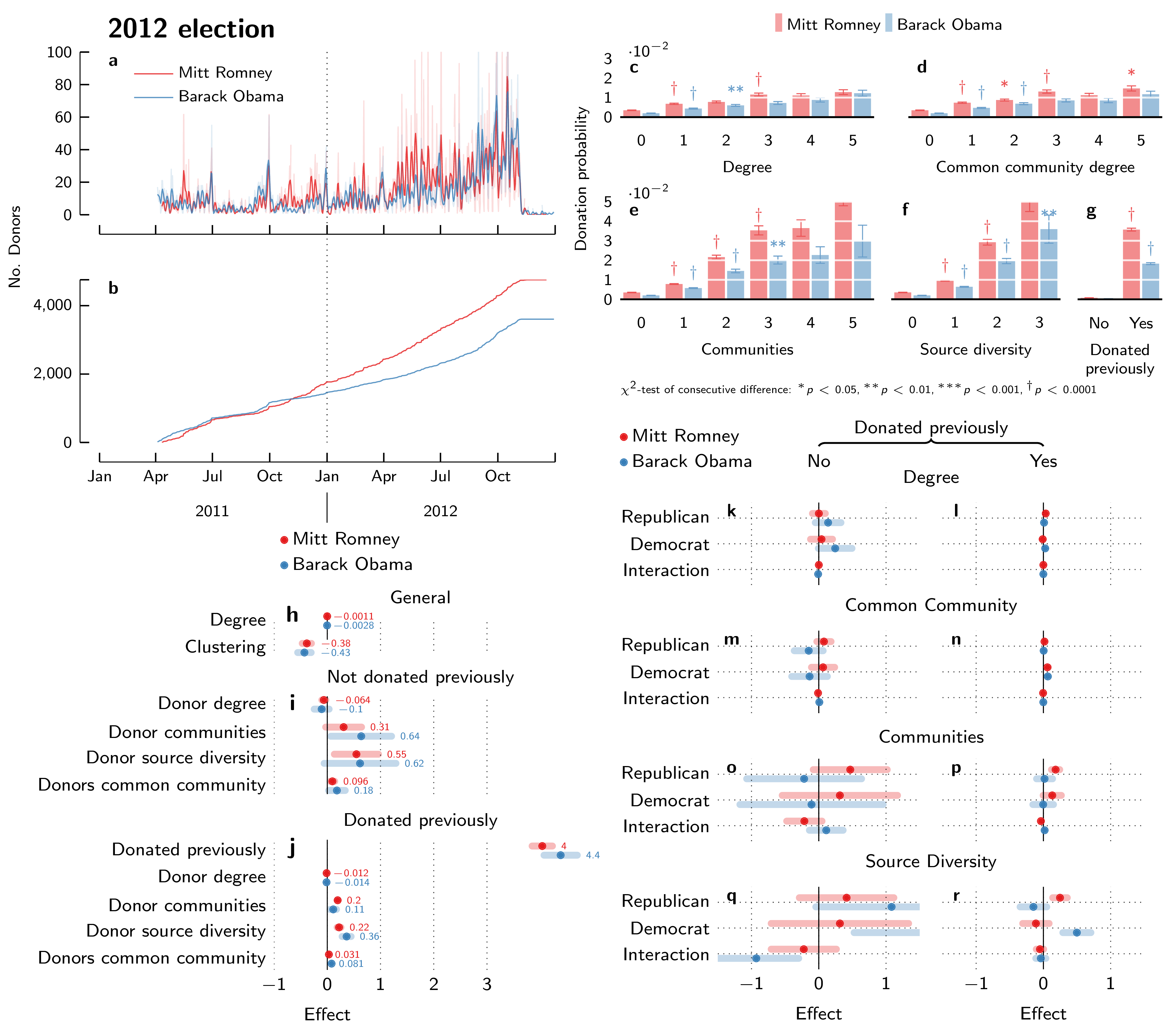

Supplement: S7 Fig — Daily donors (a)—raw data is transparent, smoothed data is solid— and cumulative donors (b), probability effect of (c) donor degree, (d) common community donor degree, (e) donor communities, (f) source diversity and (g) previous donation. Logistic regression results (h) general effects, (i) network effects for new donors, and (j) network effects for old donors. Logistic regression result for cross-cutting effects, distinguished by old/new donors for donor degree (k)–(l), common community donor degree (m)–(n), donor communities (o)–(p), and source diversity (q)–(r). Error bars show 95% confidence intervals for the coefficients. (TIFF) [file pone.0153539.s007.tiff]

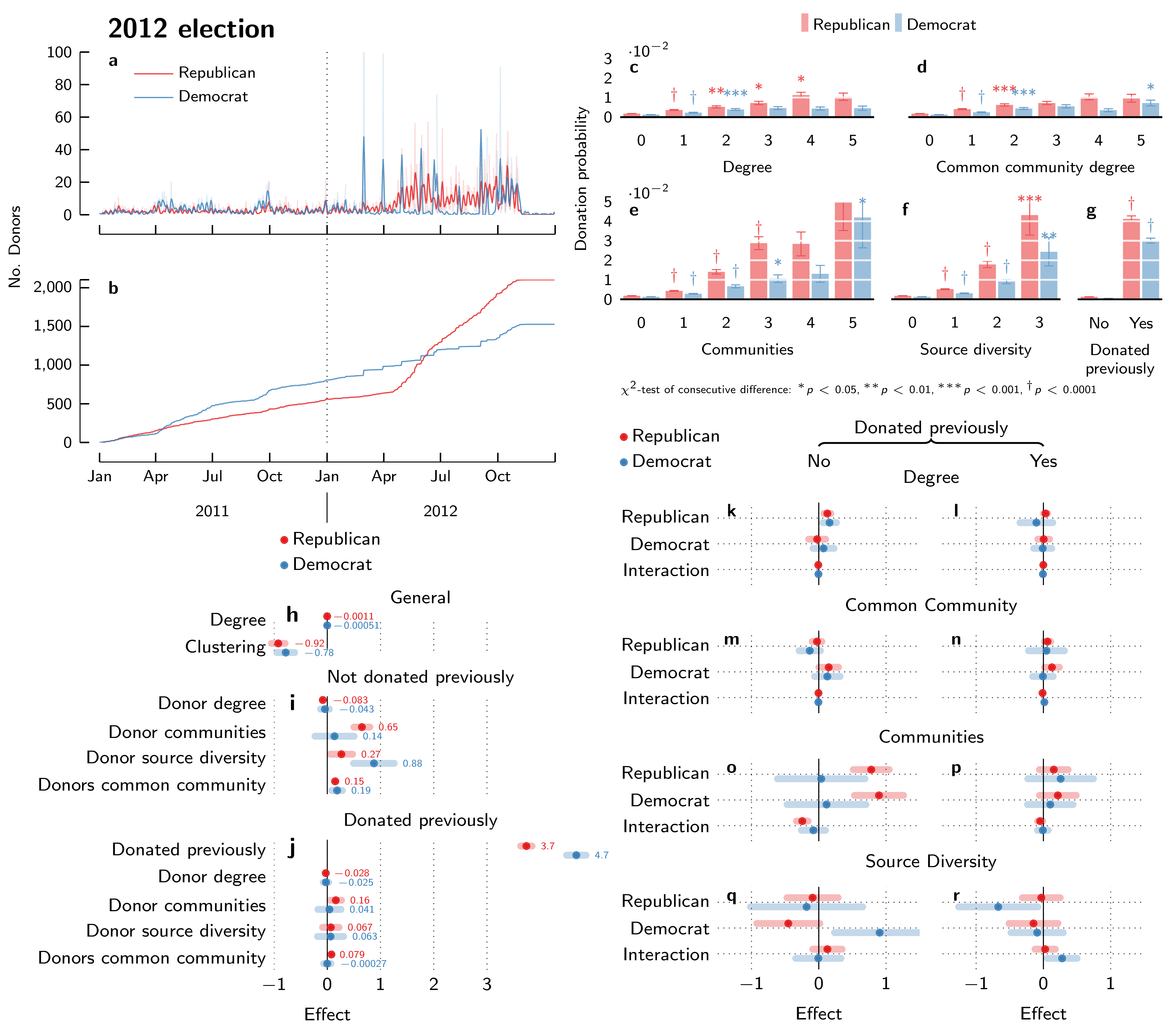

Supplement: S8 Fig — Daily donors (a)—raw data is transparent, smoothed data is solid— and cumulative donors (b), probability effect of (c) donor degree, (d) common community donor degree, (e) donor communities, (f) source diversity and (g) previous donation. Logistic regression results (h) general effects, (i) network effects for new donors, and (j) network effects for old donors. Logistic regression result for cross-cutting effects, distinguished by old/new donors for donor degree (k)–(l), common community donor degree (m)–(n), donor communities (o)–(p), and source diversity (q)–(r). Error bars show 95% confidence intervals for the coefficients. (TIFF) [file pone.0153539.s008.tiff]

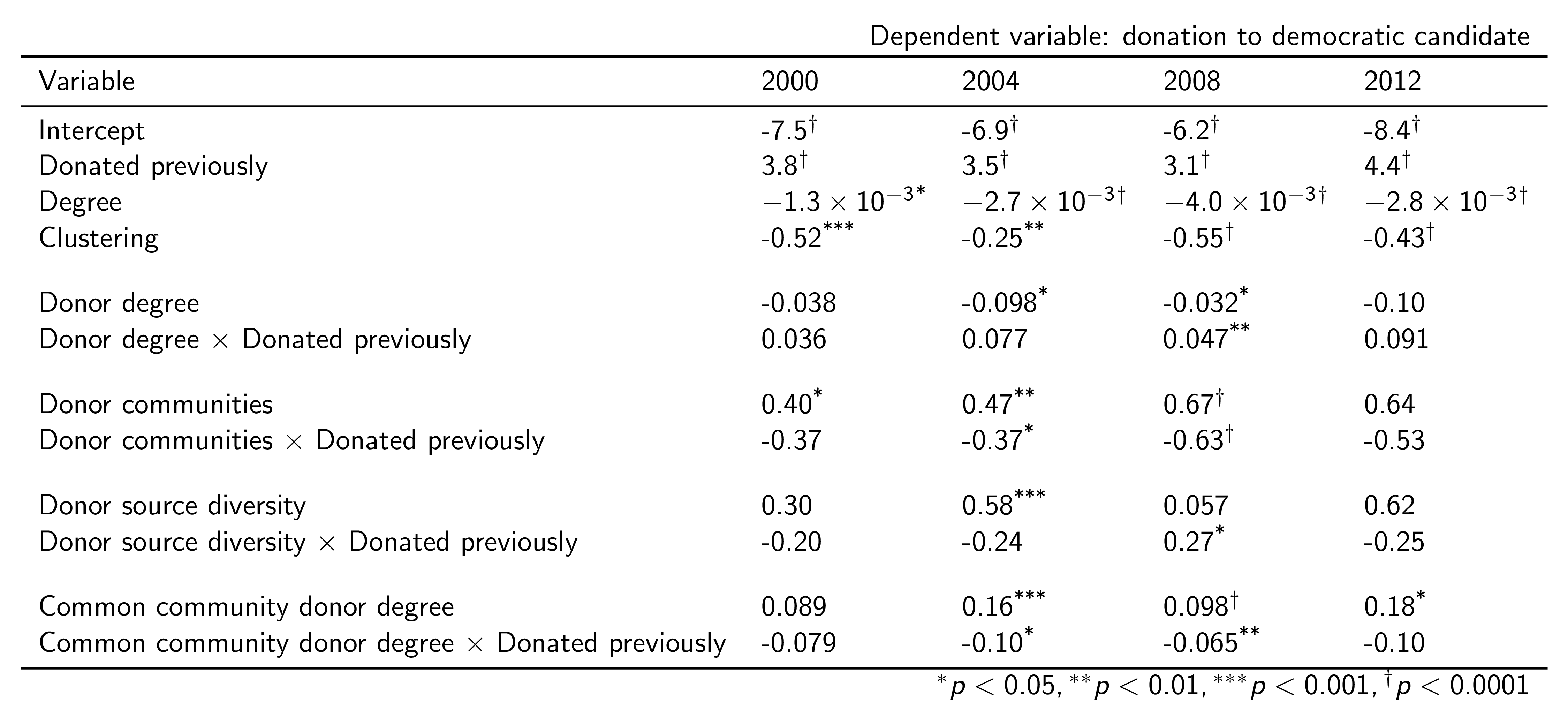

Supplement: S1 Table — Results for 2000–2012 for donation to the democratic candidate as dependent variable. (TIFF) [file pone.0153539.s009.tiff]

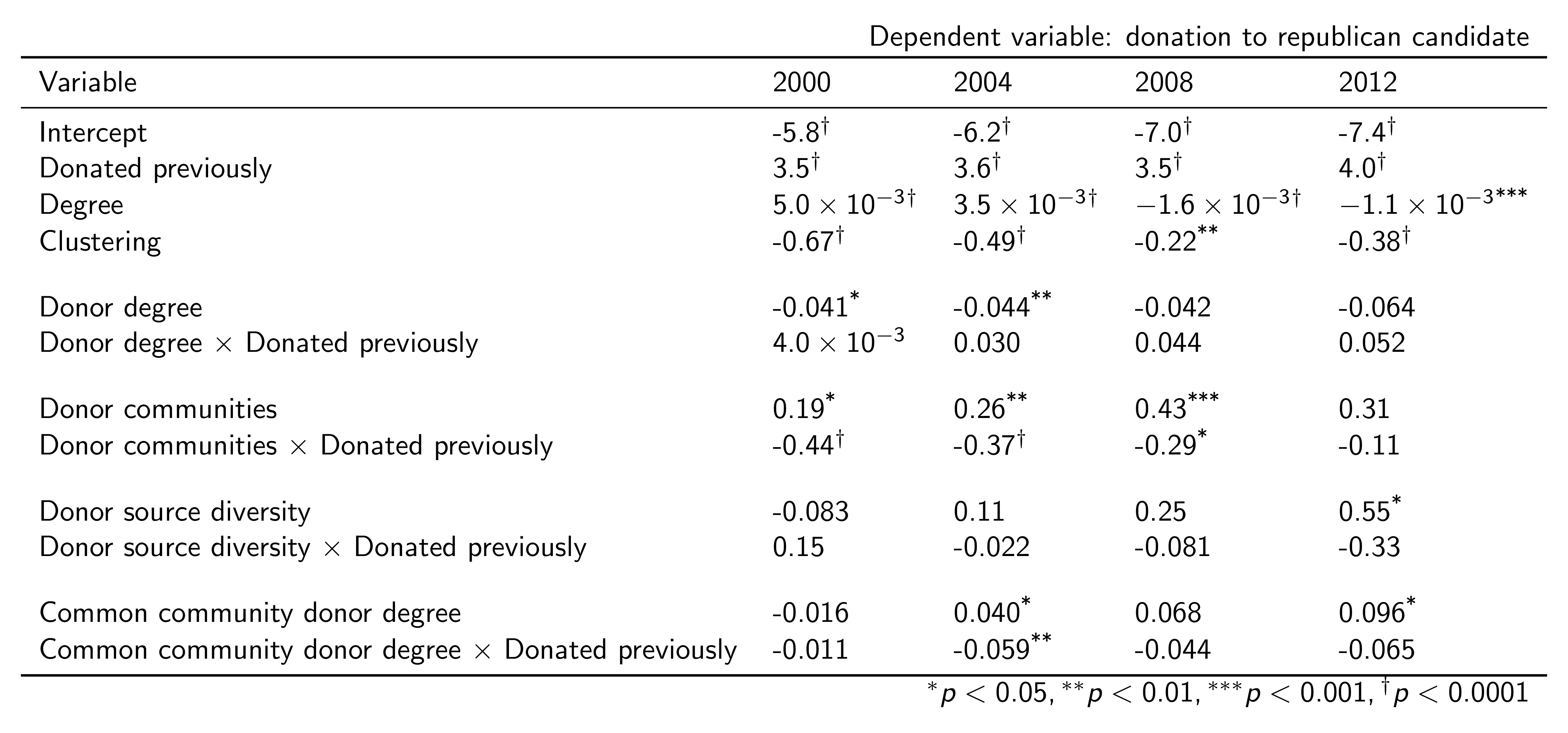

Supplement: S2 Table — Results for 2000–2012 for donation to the republican candidate as dependent variable. (TIFF) [file pone.0153539.s010.tiff]

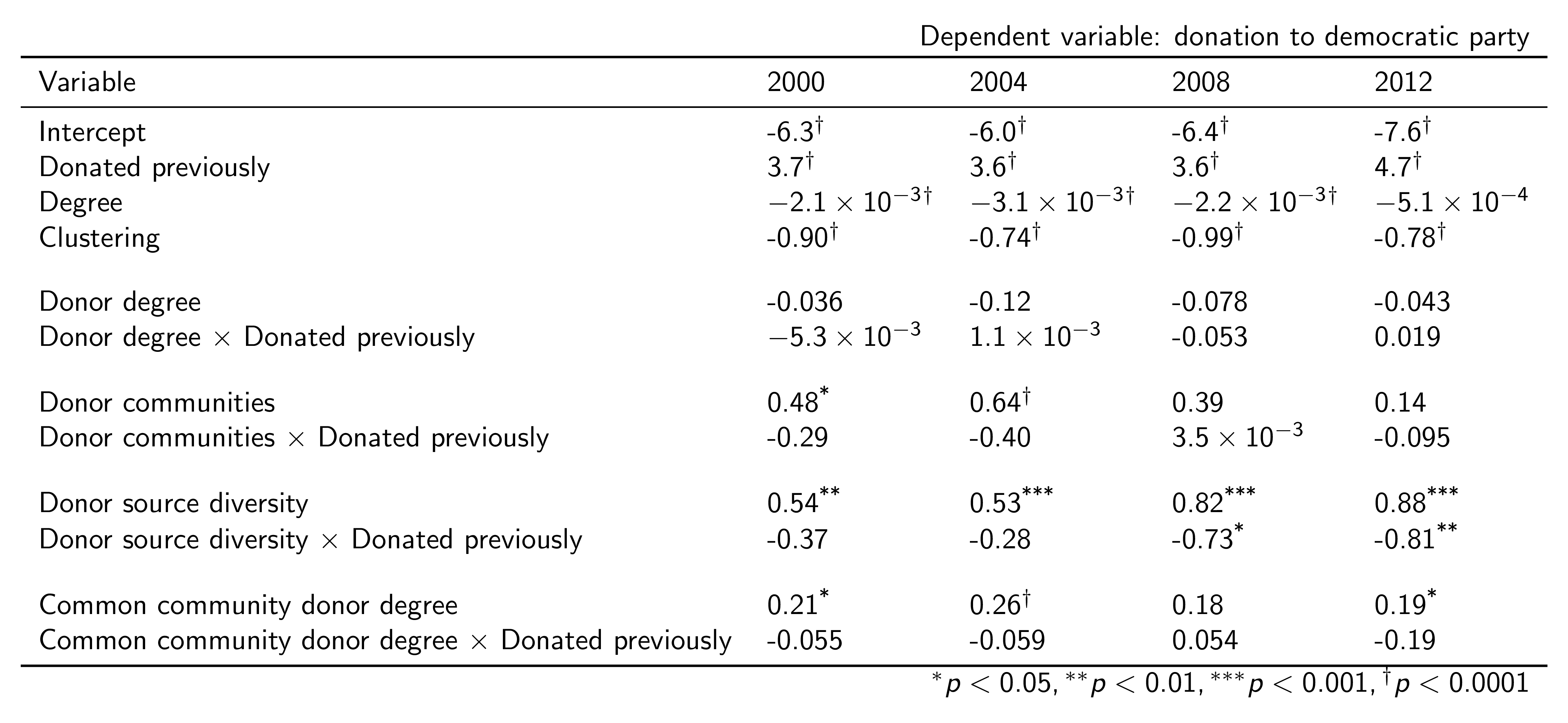

Supplement: S3 Table — Results for 2000–2012 for donation to the democratic party as dependent variable. (TIFF) [file pone.0153539.s011.tiff]

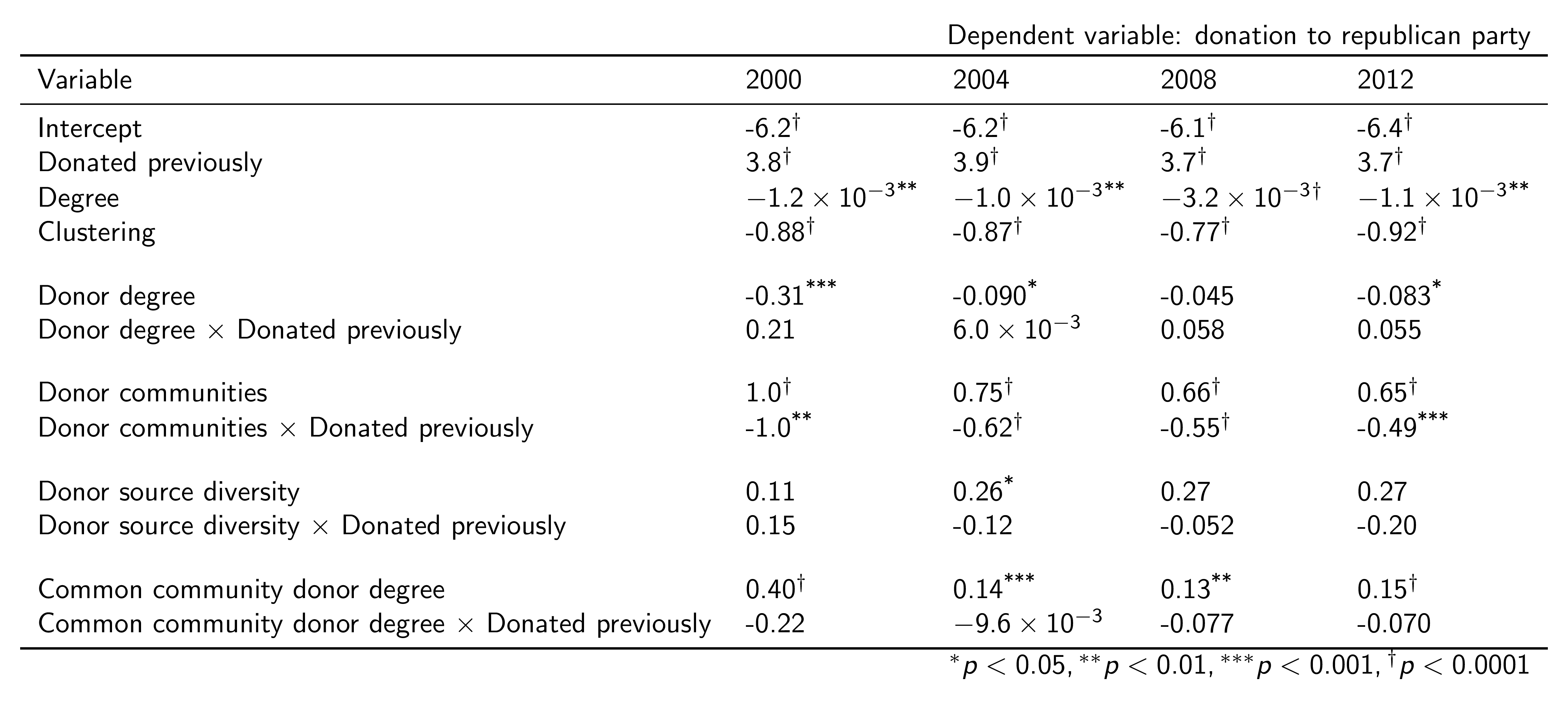

Supplement: S4 Table — Results for 2000–2012 for donation to the republican party as dependent variable. (TIFF) [file pone.0153539.s012.tiff]

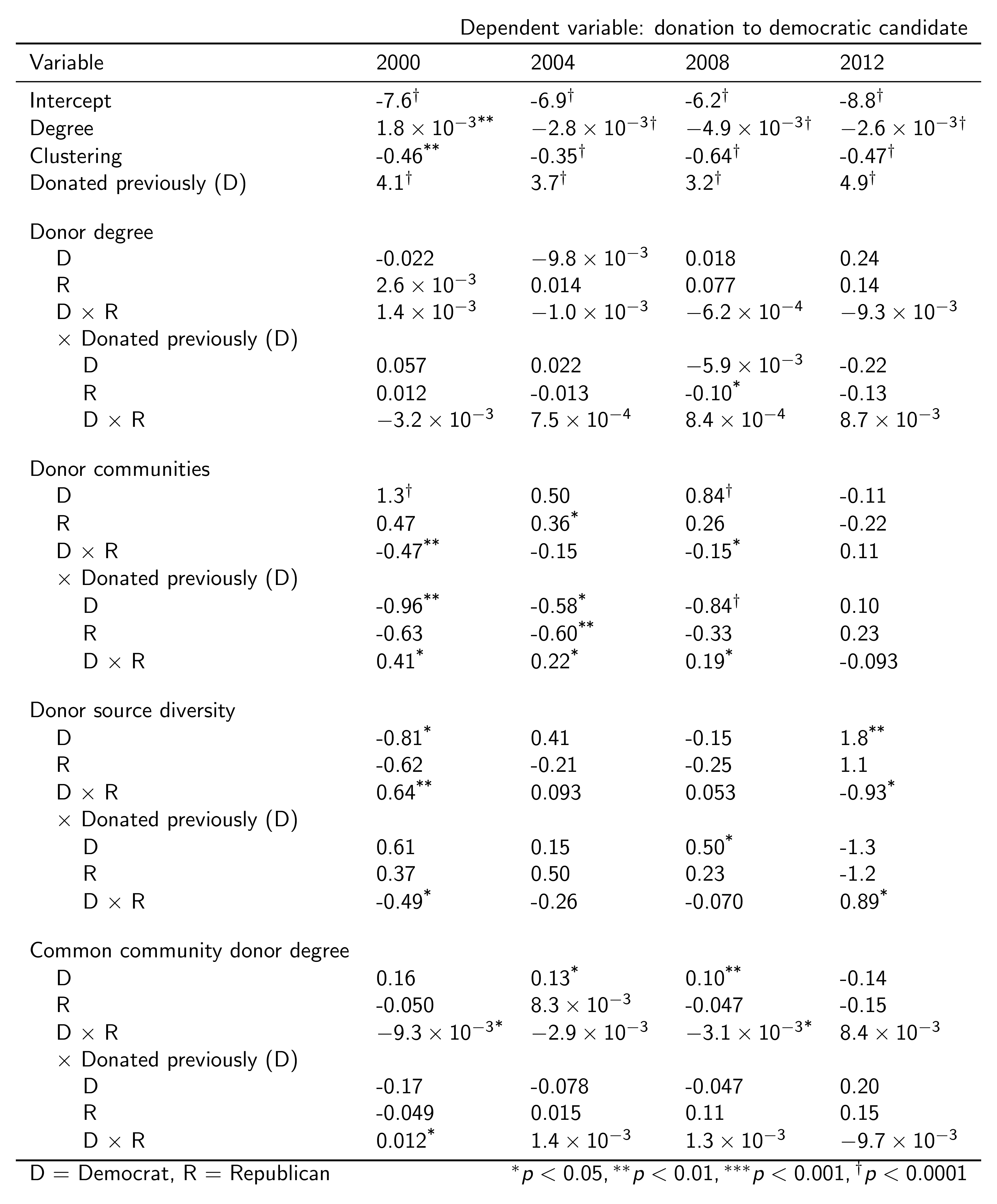

Supplement: S5 Table — Results for 2000–2012 for donation to the democratic candidate as dependent variable, including cross-exposure effects (i.e. effect of exposure to republican donors on democratic donations). (TIFF) [file pone.0153539.s013.tiff]

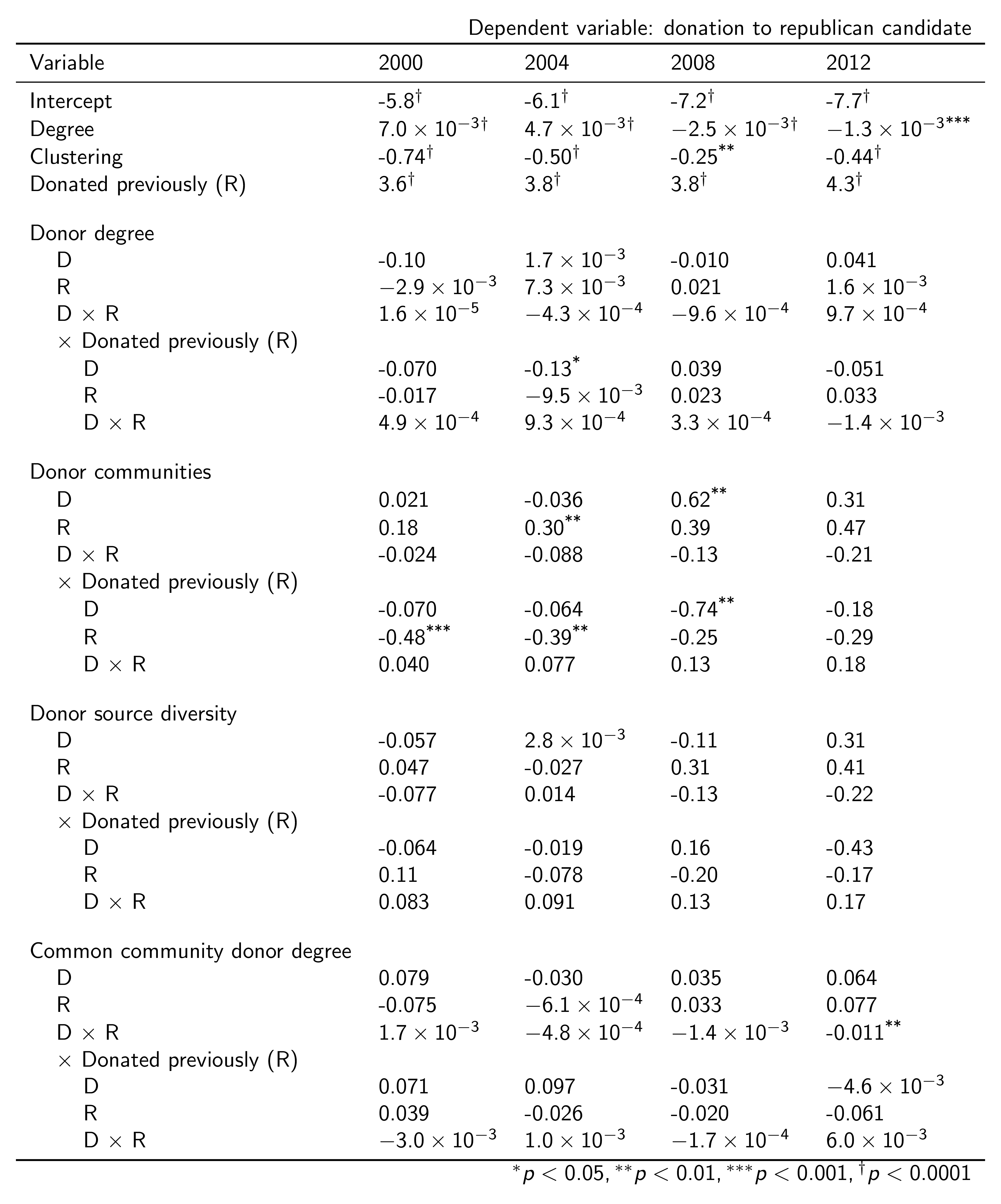

Supplement: S6 Table — Results for 2000–2012 for donation to the republican candidate as dependent variable, including cross-exposure effects (i.e. effect of exposure to democratic donors on republican donations). (TIFF) [file pone.0153539.s014.tiff]

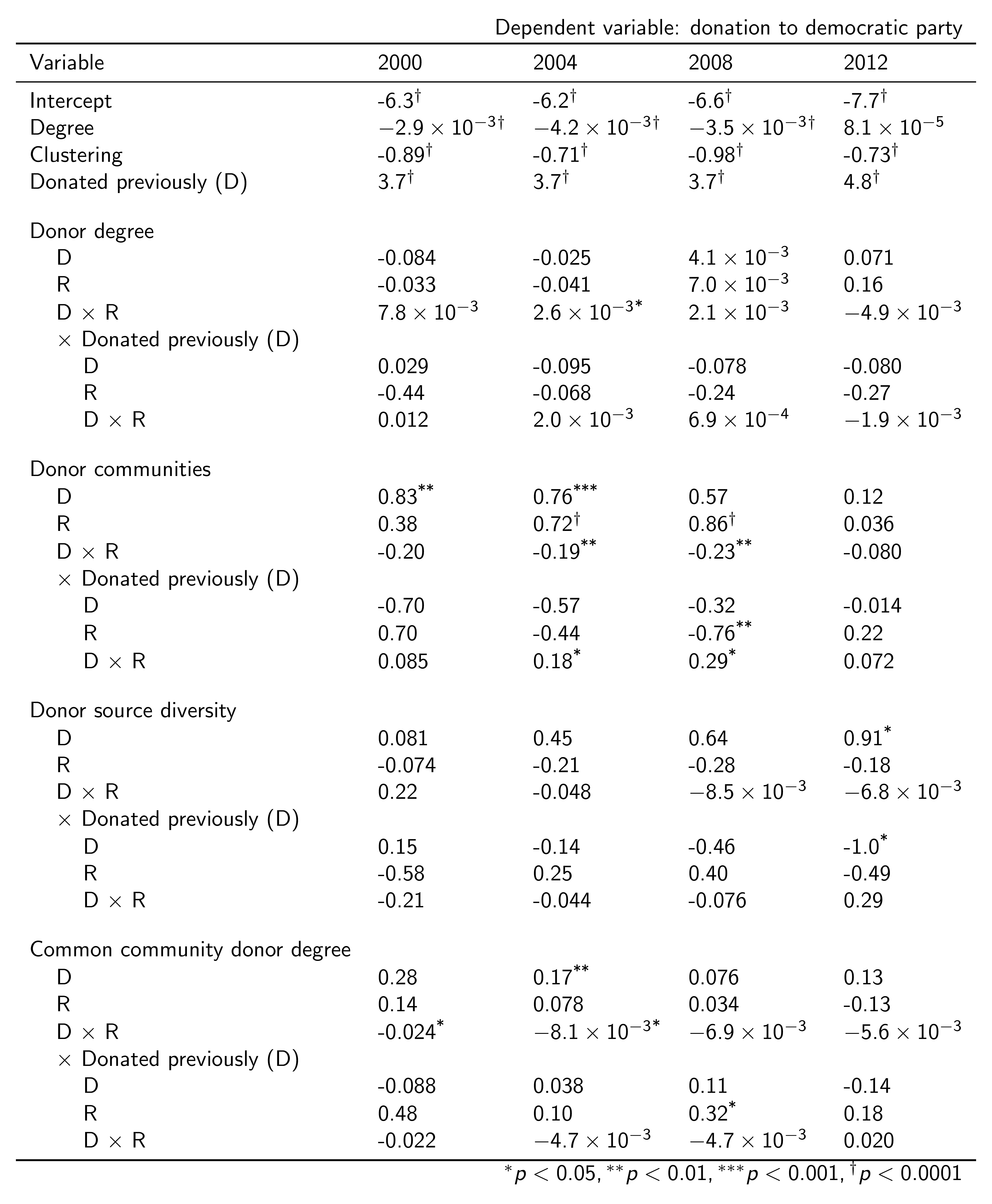

Supplement: S7 Table — Results for 2000–2012 for donation to the democratic party as dependent variable, including cross-exposure effects (i.e. effect of exposure to republican donors on democratic donations). (TIFF) [file pone.0153539.s015.tiff]

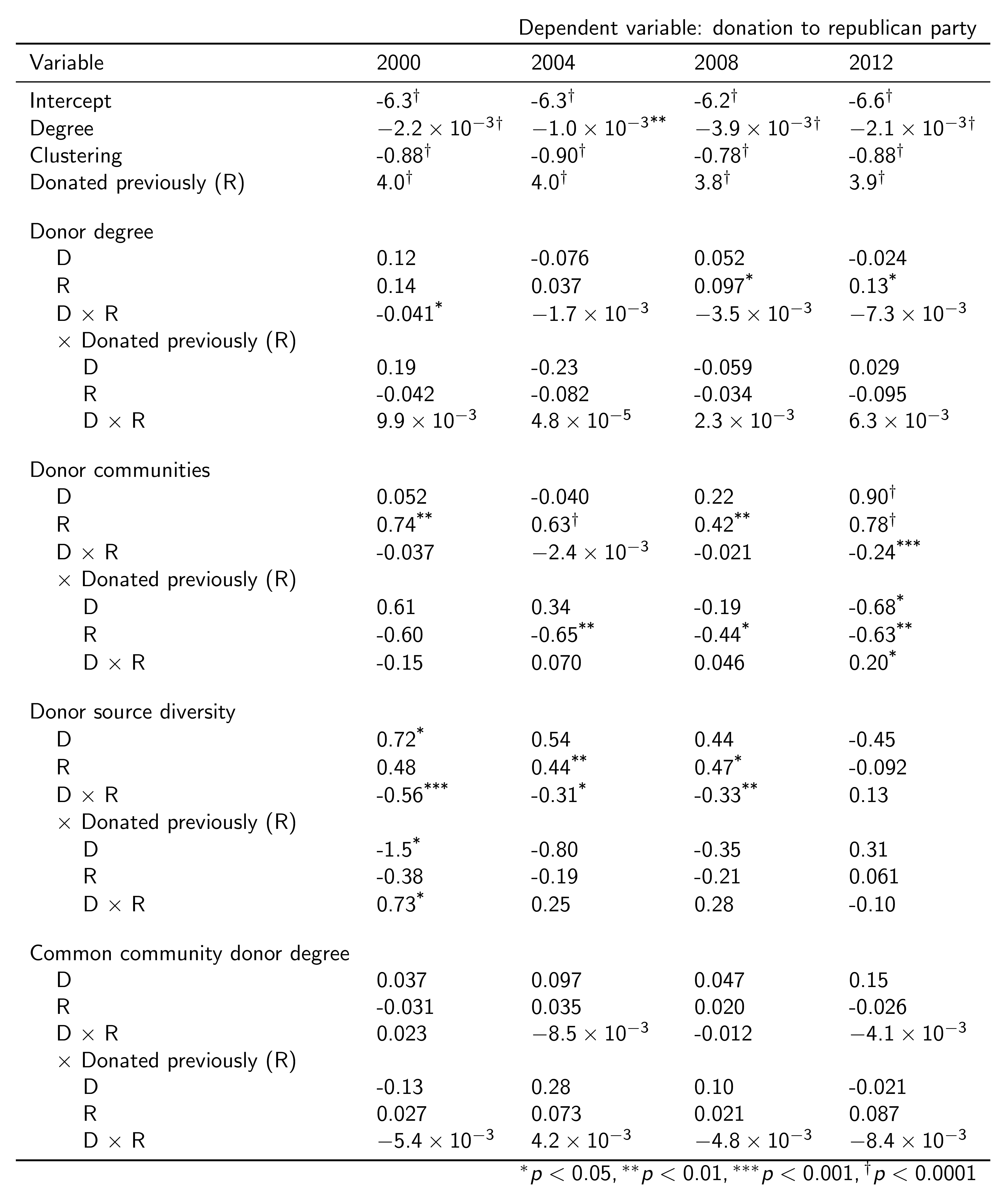

Supplement: S8 Table — Results for 2000–2012 for donation to the republican party as dependent variable, including cross-exposure effects (i.e. effect of exposure to democratic donors on republican donations). (TIFF) [file pone.0153539.s016.tiff]
